# Using the health belief model to explore nursing students’ relationships between COVID-19 knowledge, health beliefs, cues to action, self-efficacy, and behavioral intention: A cross-sectional survey study

**DOI:** 10.1097/MD.0000000000025210

**Published:** 2021-03-19

**Authors:** Fu-Ju Tsai, Yih-Jin Hu, Cheng-Yu Chen, Chie-Chien Tseng, Gwo-Liang Yeh, Jin-Fong Cheng

**Affiliations:** a Department of Nursing, Fooyin University; b Department of Health Promotion and Health Education, National Taiwan Normal University; c Department of Nursing, Registered Nurse, Mennonite Christian Hospital, Taiwan ROC.

**Keywords:** behavioral intention, COVID-19, health beliefs model, nursing students, self-efficacy

## Abstract

Nursing educators should equip nursing students with sufficient knowledge about coronavirus disease 2019 (COVID-19), perceived susceptibility, perceived severity, perceived benefits, perceived barriers, cues to action, self-efficacy, and behavioral intention in order to prevent the spread of COVID-19.

The purpose of this study was to use the health belief model to elucidate nursing students’ relationships between knowledge about COVID-19, perceived susceptibility, perceived severity, perceived benefits, perceived barriers, cues to action, self-efficacy, and behavioral intention.

A cross-sectional survey design was adopted and purposive sampling was utilized. A total of 361 nursing students participated in the study. Quantitative analysis was employed for all data analysis.

The findings showed that the nursing students had the following mean scores on knowledge of COVID-19 9.43 [standard deviation (SD)1.19], perceived susceptibility 19.41 (SD2.68), perceived severity 20.31 (SD 4.09), perceived benefits 26.52 (SD 4.08), perceived barriers 15.17 (SD5.88), cues to action 3.30 (SD1.70), self-efficacy 17.68 (SD2.83), and behavioral intention 18.46 (SD2.33). Nursing students’ demographic background, knowledge of COVID-19, perceived susceptibility, perceived severity, perceived benefits, perceived barriers, cues to action, and self-efficacy explained 58.1% of the variance in behavioral intention (*R*^*2*^ = 0.581, *F* = 29.775, *P* < .001).

Nursing educators can increase nursing students’ knowledge of COVID-19, perceived susceptibility, perceived severity, perceived benefits, perceived barriers, cues to action, and self-efficacy as effective means of health promotion to improve their behavioral intention to prevent the spread of COVID-19.

## 1 Introduction

Since the occurrence of the coronavirus disease (COVID-19) in Wuhan, Hubei Province, China on December 1, 2019, many of millions of people globally have experienced deleterious effects. COVID-19 has a very high risk of human-to-human transmission,^[1,2]^ is widely diagnosed (14,012,449 people worldwide; 454 people in Taiwan), can be both severe and fatal (mortality 596,158 people worldwide 4.25%; 7 people in Taiwan),^[3]^ and causes serious physical, psychological, emotional, and social trauma.^[4–6]^ Relevant knowledge about COVID-19,^[7]^ and related health beliefs, self-efficacy, and preventive behaviors, is critically important to decrease the rate of infection, reduce the mortality rate, and maintain peoples’ health and quality of life.^[8–10]^

COVID-19 is an atypical coronavirus, with an incubation period of 1 to 14 day or a delay of several weeks to several months. It constitutes a human-to-human droplet and contact infection.^[11–14]^ It is also possible to develop COVID-19 with symptoms or asymptomatically.^[15,16]^ Symptoms may include chest tightness, headache, difficulty breathing, fever >38°C, sneezing, runny nose, stuffy nose, cough, nausea, abdominal pain, diarrhea, muscle ache, fatigue, and general weakness, among others.^[17–21]^ Furthermore, people commonly experience major associated physical, psychological, emotional, and social issues, including insomnia, stress, anxiety, depression, social support and coping problems, well-being issues, and burnout in daily life.^[22,23]^ In addition to the need for people to be cognizant of these symptoms and related impacts of COVID-19, it is also urgent that people understand the demonstrated effectiveness of certain personal self-preventive measures, such as frequently washing one's hands, measuring one's body temperature, wearing a mask,^[24]^ avoiding entering and exiting crowded locations, opening doors and windows to circulate air, using hand sanitizer with 60% to75% alcohol content, and wiping commonly used surfaces with bleach.^[17–19]^

The structure of the health belief model regarding implementing and sustaining healthy behaviors includes the following: perceived susceptibility, perceived severity, perceived benefits, perceived barriers, and cues to action. Personal behaviors and health beliefs are adopted by individuals in daily life. In order to maintain and promote peoples’ health, healthy behaviors are significant, and produce important impacts on peoples’ health beliefs to practice healthy behaviors and maintain a healthy life. People's health and diseases are related to health beliefs in terms of perceived susceptibility, severity, benefits, and barriers of the diseases, and are manifested in preventive behaviors and behavioral intention.^[25–27]^

Self-efficacy, in our context, refers to personal effectiveness in using personal health beliefs and behaviors to achieve health goals in daily life. People high in self-efficacy generally hold the belief that, through their own effects, they can achieve desired health results in an effective manner. One of the reasons why the dimension of self-efficacy is so critical in the consistent performance of healthy behaviors is that individuals’ typically face operational barriers when they attempt to perform those healthy behaviors. As a consequence, they need both self-confidence and will-power, that is, self-efficacy to persist through those barriers, reinforce their health beliefs, and implement practical actions to reach their health goals.^[25]^

Based on the growing global impact of COVID-19, the health belief model is utilized to understand the health beliefs, self-efficacy, and preventive behaviors of nursing students with respect to COVID-19. Indeed, it is hoped that the model can be employed to assess whether nursing students are actually implementing and promoting personal health beliefs, self-efficacy, and preventive behaviors in their patients’ daily lives.^[26–30]^ It is the responsibility of nursing educators to equip nursing students with thorough knowledge about COVID-19, and accurate health beliefs about the perceived susceptibility, perceived severity, perceived benefits, perceived barriers, cues to action, self-efficacy, and behavioral intention^[26–30]^ in order to prevent the spread of this infectious disease both locally and globally.

## 2 Purpose

The purpose of this study was to use the health belief model to explore nursing students’ relationships between knowledge about COVID-19, perceived susceptibility, perceived severity, perceived benefits, perceived barriers, cues to action, self-efficacy, and behavioral intention.

## 3 Methods

### 3.1 Design

A cross-sectional survey design was adopted in this study.

### 3.2 Framework

**Figure 1:**
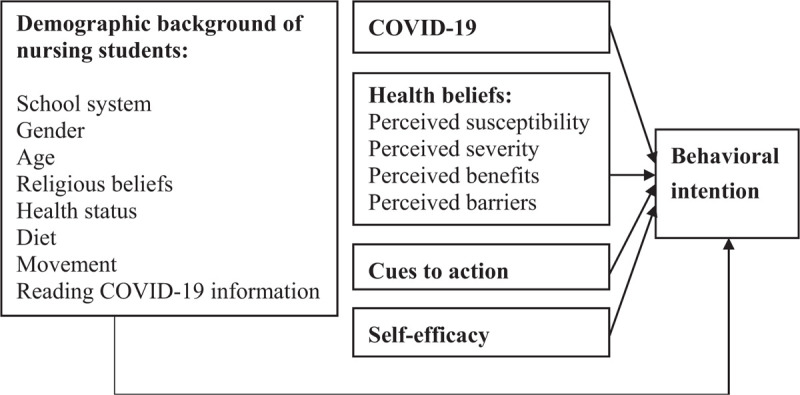
Indicated the demographic background of nursing students, COVID-19, health beliefs, cues to action, and self-efficacy to predict behavioral intention.

The framework of this study was constructed to survey the demographic background of nursing students in terms of school system, gender, age, religious beliefs, health status, diet, movement, and reading of COVID-19 information, in relation to COVID-19 knowledge, as well as the relationships between the health beliefs model on perceived susceptibility, perceived severity, perceived benefits, perceived barriers, cues to action, self-efficacy, and behavioral intention (Fig. 1).

### 3.3 Participants

According to Krejcie and Morgan, the samples of the study were required 346 nursing students.^[31]^ The study was no criteria in inclusion and exclusion. A total of 361 nursing students participated in the survey study. Purposive sampling was used in the study. The 361 nursing students comprised, at the university level: 200 in a 5-year program; 45 in a 4-year program; and 116 in a 2-year program.

### 3.4 Ethical considerations

This survey study of nursing students was authorized by the Institutional Review Board / Ethics Committee of Mennonite Christian Hospital (IRB No. 20–04-008) in Taiwan, ROC. All nursing students participated voluntarily in answering the questionnaires about demographic background, cues to action, COVID-19 knowledge, perceived susceptibility, perceived severity, perceived benefits, perceived barriers, self-efficacy, and behavioral intention.

### 3.5 Instruments

The instrument of this study was self-designed and used a health belief model^[25,26,27]^ constructed by the authors. The questionnaire included nursing students’ demographic background in terms of school system, gender, age, religious beliefs, health status, diet, movement, reading COVID-19 information, cues to action (television, broadcast, news, network, health care workers, poster promotion, school promotion, promotional car, and others). The questionnaire comprised 42-items, containing: knowledge about COVID-19 (items 1–11), and relationships between the health beliefs model on perceived susceptibility (items 12–16), perceived severity (items 17–22), perceived benefits (items 23–28), perceived barriers (items 29–34), self-efficacy (items 35–38), and behavioral intention (items 39–42). Items 1 to 11 on the questionnaire were answered by “true” and “false” responses, while a 5-point Likert-type scale was used for items 12 to 42. The content validity index of the questionnaire on the 7-part measure, including COVID-19 knowledge, perceived susceptibility, perceived severity, perceived benefits, perceived barriers, self-efficacy, and behavioral intention was 0.85 to 0.92 as established by 5 experts. The reliabilities of the preliminary test on the 7-part measure (n = 83) were as follows: COVID-19 knowledge had a Kuder–Richardson reliability−20 of 0.38; perceived susceptibility had a Cronbach α of 0.71; perceived severity had a Cronbach α of 0.84; perceived benefits had a Cronbach α of 0.94; perceived barriers had a Cronbach α of 0.92; self-efficacy had a Cronbach α of 0.96; and behavioral intention had a Cronbach α of 0.96. The reliabilities of the 7-part measure (n = 361) were as follows: COVID-19 knowledge had a Kuder–Richardson reliability−20 of 0.46; perceived susceptibility had a Cronbach α of 0.61; perceived severity had a Cronbach α of 0.84; perceived benefits had a Cronbach α of 0.96; perceived barriers had a Cronbach α of 0.91; self-efficacy had a Cronbach α of 0.96; and behavioral intention had a Cronbach α of 0.96.

### 3.6 Data collection

The researcher administered a questionnaire to 400 nursing students, and explained that the questionnaire was used to survey nursing students’ demographic background, cues to action, and relationships between COVID-19 knowledge, perceived susceptibility, perceived severity, perceived benefits, perceived barriers, self-efficacy, and behavioral intention in a university. Each of the 400 nursing students could decide to fill out the survey questionnaires completely or incompletely. Nursing students self-responded to the 42-item questions regarding COVID-19 knowledge, perceived susceptibility, perceived severity, perceived benefits, perceived barriers, self-efficacy, and behavioral intention. 361 (90.25%) of the questionnaires were completely finished by nursing students and 39 (9.75%) of the questionnaires were either incomplete or unreturned. The researcher collected a total of 361 questionnaires from May 25 to May 29, 2020.

### 3.7 Data analysis

Quantitative analysis was employed for all data analysis. The Statistical Package for the Social Sciences 23.0 statistical package was used to analyze all data in the research. The data analysis of the study included percentages, frequencies, means, standard deviations (SDs), single sample *t* test, Spearman rho correlation, and multiple hierarchical regression analysis.

## 4 Results

### 4.1 Demographic background

Nursing students included 200 (55.40%) in a 5-year program, 45 (12.50%) in a 4-year program, and 116 (32.10%) in a 2-year program (Table 1). Concerning gender distribution, 39 (10.80%) were males and 322 (89.20%) were females (Table 1). In terms of age distribution, the nursing students included 216 (59.80%) 20-years-old and 145 (40.20%) who were above 20-years-old (Table 1). Regarding religious beliefs, there were 178 (49.30%) nursing students with no religious beliefs and 183 (50.70%) with religious beliefs (Table 1). Concerning health status, 193 (53.50%) nursing students had an ordinary health status and 168 (46.50%) had a very good health status (Table 1). In terms of diet, 117 (32.40%) nursing students ate a normal diet without in 3 meals and 244 (67.60%) ate a normal diet in 3 meals (Table 1). Regarding movement, 259 (71.70%) nursing students had no regular movement, and 102 (28.30%) had regular movement (Table 1). Concerning reading COVID-19 information, 55 (15.20%) of the nursing students had done no reading and 306 (84.80%) had done some reading (Table 1).

**Table 1:** Demographic background of nursing students.

N = 361	Items	Frequency	Percentage
School system	1. 5-year program	200	55.40%
	2. 4-year program	45	12.50%
	3. 2-year program	116	32.10%
Gender	1. Male	39	10.80%
	2. Female	322	89.20%
Age	1. 20-years-old	216	59.80%
	2. Above 20-years-old	145	40.20%
Religious beliefs	1. No religious beliefs	178	49.30%
	2. Religious beliefs	183	50.70%
Health status	1. Ordinary health status	193	53.50%
	2. Very good health status	168	46.50%
Diet	1. Normal diet without 3 meals	117	32.40%
	2. Normal diet in 3 meals	244	67.60%
Movement	1. No regular movement	259	71.70%
	2. Regular movement	102	28.30%
Reading COVID-19 information	1. No reading	55	15.20%
	2. Reading	306	84.80%

### 4.2 Cues to action for nursing students

In the study, nursing students’ cues to action included 353 (97.80%) from television, 56 (15.50%) broadcast, 82 (22.70%) news, 227 (62.90%) network, 142 (39.30%) health care workers, 105 (29.10%) poster promotion, 199 (55.10%) school promotion, 24 (6.60%) promotional car, and 3 (0.80%) others (Table 2).

**Table 2:** Cues to action for nursing students.

N = 361	Items	Frequency	Percentage
Cues to action	1. Television	353	97.80%
	2. Broadcast	56	15.50%
	3. News	82	22.70%
	4. Network	227	62.90%
	5. Health care workers	142	39.30%
	6. Poster promotion	105	29.10%
	7. School promotion	199	55.10%
	8. Promotional car	24	6.60%
	9. Others	3	0.80%

### 4.3 Nursing student's mean scores

The results of the study showed that the nursing students had the following mean scores on knowledge of COVID-19 9.43 (SD1.19); perceived susceptibility 19.41 (SD2.68); perceived severity 20.31 (SD 4.09); perceived benefits 26.52 (SD 4.08); perceived barriers 15.17 (SD5.88); cues to action 3.30 (SD1.70); self-efficacy 17.68 (SD2.83); and behavioral intention 18.46 (SD2.33) (Table 3).

**Table 3:** Nursing student's mean scores.

N = 361	Items	Mean	SD
Knowledge	11	9.43	1.19
Perceived susceptibility	5	19.41	2.68
Perceived severity	6	20.31	4.09
Perceived benefits	6	26.52	4.08
Perceived barriers	6	15.17	5.88
Cues to action	9	3.30	1.70
Self-efficacy	4	17.68	2.83
Behavioral intention	4	18.46	2.33

### 4.4 Spearman rho correlation analysis results

Spearman rho correlation analysis results are presented in Table 4. It was found that 361 nursing students’ COVID-19 knowledge was positively correlated with self-efficacy, *r* = .162 (*P* < .01), and behavioral intention, *r* = .158 (*P* < .01). In addition, nursing students’ perceived susceptibility was positively correlated with perceived severity, *r* = .279 (*P* < .01), perceived benefits, *r* = .325 (*P* < .01), self-efficacy, *r* = .236 (*P* < .01), and behavioral intention, *r* = .279 (*P* < .01). Moreover, nursing students’ perceived severity was positively correlated with perceived benefits, *r* = .158 (*P* < .01), and perceived barriers, *r* = .193 (*P* < .01). Furthermore, nursing students’ perceived benefits were positively correlated with perceived barriers, *r* = −.237 (*P* < .01), self-efficacy, *r* = .451 (*P* < .01), and behavioral intention, *r* = .525 (*P* < .01). Nursing students’ perceived barriers were positively correlated with self-efficacy, *r* = −.224 (*P* < .01), and behavioral intention, *r* = −.352 (*P* < .01). Nursing students’ cues to action were also positively correlated with self-efficacy, *r* = .104 (*P* < .05). Finally, nursing students’ self-efficacy was positively correlated with behavioral intention, *r* = .667 (*P* < .01).

**Table 4:** Spearman rho correlation analysis results.

N = 361	COVID-19 knowledge	Perceived susceptibility	Perceived severity	Perceived benefits	Perceived barriers	Cues to action	Self-efficacy	Behavioral intention
COVID-19 knowledge	1							
Perceived susceptibility	.084	1						
Perceived severity	.092	.279^∗∗^	1					
Perceived benefits	.068	.325^∗∗^	.158^∗∗^	1				
Perceived barriers	−.020	.034	.193^∗∗^	−.237^∗∗^	1			
Cues to action	.026	−.019	.053	.051	−.092	1		
Self-efficacy	.162^∗∗^	.236^∗∗^	.075	.451^∗∗^	−.224^∗∗^	.104^∗^	1	
Behavioral intention	.158^∗∗^	.279^∗∗^	.067	.525^∗∗^	−.352^∗∗^	.094	.667^∗∗^	1

### 4.5 Multiple hierarchical regression analysis results

In model 1, nursing students’ demographic background regarding school system, gender, age, religious beliefs, health status, diet, movement, and reading COVID-19 information explained 5.2% of the variance in behavioral intention (*R*^*2*^ = 0.052, *F* = 2.118, *P* < .05) (Table 5). Table 5 shows the data on the health status (*B* = 0.210, *t* = 3.706, *P* < .001), and it can be seen that nursing students had a very good health status with the highest impact on their behavioral intention.

**Table 5:** Multiple hierarchical regression analysis results.

	Model 1		Model 2	
	Standardized coefficients	*t* values	Standardized coefficients	*t* values
Variables	Beta distribution		Beta distribution	
Constant		67.695^∗∗∗^		5.446^∗∗∗^
School system				
Four-year program	−.004	−.050	.012	.209
Two-year program	.031	.337	−.008	−.131
Gender				
male	−.078	−1.471	−.001	−.037
Age				
Above 20-years-old	−.037	−1.368	−.033	−.492
Religious beliefs				
No religious beliefs	−.065	−1.244	−.011	−.309
Health status				
Very good health status	.210	3.706^∗∗∗^	.059	1.560
Diet				
Normal diet without 3 meals	−.004	−.069	−.012	−.323
Movement				
Regular movement	−.003	−.046	−.022	−.590
Reading COVID-19 information				
No reading	−.018	−.337	.072	1.967
COVID-19 knowledge			.047	1.290
Perceived susceptibility			.093	2.315^∗^
Perceived severity			−.007	−.193
Perceived benefits			.274	6.446^∗∗∗^
Perceived barriers			−.151	−4.008^∗∗∗^
Cues to action			.044	1.203
Self-efficacy			.480	11.513^∗∗∗^
	*R*^*2*^ = 0.052	*F* = 2.118^∗^	*R*^*2*^ = 0.581	*F* = 29.775^∗∗∗^
			Δ*R*^*2*^ = 0.529	Δ*F* = 62.019^∗∗∗^

In model 2, the results of this study showed that nursing students’ demographic background regarding school system, gender, age, religious beliefs, health status, diet, movement, reading COVID-19 information, COVID-19 knowledge, perceived susceptibility, perceived severity, perceived benefits, perceived barriers, cues to action, and self-efficacy explained 58.1% of the variance in behavioral intention (*R*^*2*^ = 0.581, *F* = 29.775, *P* < .001) (Table 5).

Nursing students’ demographic background concerning school system, gender, age, religious beliefs, health status, diet, movement, and reading COVID-19 information were controlled, COVID-19 knowledge, perceived susceptibility, perceived severity, perceived benefits, perceived barriers, cues to action, and self-efficacy explained 52.9% of the variance in behavioral intention (*R*^2^ = 0.529, Δ*F* = 62.019, *P* < .001) (Table 5).

Table 5 presents the data on perceived susceptibility (*B* = 0.093, *t* = 2.315, *P* < .05), perceived benefits (*B* = 0.274, *t* = 6.446, *P* < .001), perceived barriers (*B* = 0.151, *t* = −4.008, *P* < .001), and self-efficacy (*B* = 0.480, *t* = 11.513, *P* < .001). It can be seen that nursing students’ high self-efficacy, high perceived benefits, low perceived barriers, and high perceived susceptibility had the highest sequential impact on their behavioral intention.

## 5 Discussion

According to the World Health Organization, COVID-19 is currently an epidemic and infective disease,^[32]^ is inflicting serious damage on people physically, psychologically, emotionally, and socially,^[33]^ and threatens the world economy.^[34]^ Since many millions of people are suffering from COVID-19 globally, effective preventing measures are critical. Washing one's hands and wearing a face mask are demonstrably correlated with a lower rate of spread of COVID-19, and constitute effective ways to prevent the infectious disease among community residents.^[35,36]^ COVID-19 preventive measures are major components of health literacy in health promotion.^[37,38]^ This study aimed to elucidate nursing students’ COVID-19 knowledge, health beliefs, self-efficacy, and behavioral intention.

The findings indicated that nursing students’ cues to action included television, broadcast, news, network, health care workers, poster promotion, school promotion, promotional car, and others. The results of this study demonstrated that television is the best communication tool, network is the second-best, and school promotion is the third-best for nursing students concerning COVID-19 preventive measures.

In addition, nursing students’ mean scores were as follows: COVID-19 knowledge, perceived susceptibility, perceived severity, perceived benefits, perceived barriers, cues to action, self-efficacy, and behavioral intention. All nursing students’ health beliefs associated with all items on the mean scores indicated good healthy beliefs concerning COVID-19 knowledge, perceived susceptibility, perceived severity, perceived benefits, perceived barriers, cues to action, self-efficacy, and behavioral intention.

From the Spearman rho correlations analysis, all nursing students’ COVID-19 knowledge was positively correlated with health beliefs, self-efficacy, and behavioral intention. The findings also demonstrated that all nursing students possessed COVID-19 knowledge, evaluated their health beliefs, increased individuals’ self-efficacy, and practiced behavioral intention for preventing the spread of COVID-19.

Nursing students’ demographic background regarding school system, gender, age, religious beliefs, health status, diet, movement, reading COVID-19 information, including health beliefs about COVID-19 knowledge, perceived susceptibility, perceived severity, perceived benefits, perceived barriers, cues to action, and self-efficacy explained 58.1% of the variance in behavioral intention. In accordance with a previous study,^[39]^ practicing self-efficacy is strongly associated with behavioral intention. Our study offers the same findings as that study concerning implementing public health strategies to increase COVID-19 knowledge and health beliefs regarding physical, psychological, emotional, and social health promotion. Peoples’ positive coping, positive responses, and job satisfaction strengthen psychological prevention^[40]^ and psychological health, which are associated with physical, emotional, and social health for avoiding COVID-19 infection.^[41,42]^

## 6 Limitations

The limitations of this study are that the participants were limited to 361 nursing students in 200 five-year, 45 four-year, and 116 two-year nursing education programs at a university. This study may have limited the data that were collected all 361 nursing students. In addition, all participants were limited to nursing students in the Department of Nursing at a University in Kaohsiung City, Taiwan, ROC.

## 7 Conclusions

This study found that nursing students’ mean scores were as follows: COVID-19 knowledge 9.43, perceived susceptibility 19.41, perceived severity 20.31, perceived benefits 26.52, perceived barriers 15.17, cues to action 3.30, self-efficacy 17.68, and behavioral intention 18.46. Nursing students’ demographic background, COVID-19 knowledge, perceived susceptibility, perceived severity, perceived benefits, perceived barriers, cues to action, and self-efficacy explained 58.1% of the variance in behavioral intention. Therefore, nursing educators can increase COVID-19 knowledge, perceived susceptibility, perceived severity, perceived benefits, perceived barriers, cues to action, and self-efficacy for nursing students as effective health promotion strategies to improve their behavioral intention and prevent the spread of COVID-19 locally and globally.

## Author contributions

**Conceptualization:** Fu-Ju Tsai.

**Data curation:** Fu-Ju Tsai.

**Formal analysis:** Fu-Ju Tsai, Cheng-Yu Chen.

**Funding acquisition:** Fu-Ju Tsai.

**Investigation:** Fu-Ju Tsai.

**Methodology:** Fu-Ju Tsai, Yih-Jin Hu, Cheng-Yu Chen, Gwo-Liang Yeh.

**Project administration:** Fu-Ju Tsai, Yih-Jin Hu.

**Resources:** Fu-Ju Tsai, Yih-Jin Hu.

**Software:** Fu-Ju Tsai.

**Supervision:** Yih-Jin Hu, Cheng-Yu Chen, Chie-Chien Tseng, Gwo-Liang Yeh, Jin-Fong Cheng.

**Validation:** Yih-Jin Hu, Cheng-Yu Chen, Chie-Chien Tseng, Gwo-Liang Yeh, Jin-Fong Cheng.

**Visualization:** Yih-Jin Hu, Cheng-Yu Chen, Chie-Chien Tseng, Gwo-Liang Yeh, Jin-Fong Cheng.

**Writing – original draft:** Fu-Ju Tsai.

**Writing – review & editing:** Fu-Ju Tsai, Yih-Jin Hu.

## Corrections

When originally published, the section heading 3.1 Design was incorrectly printed as 3.1 2.1 Design. It has since been corrected.
